# Kawasaki Disease in a Young Adult: A Case Report and a Review of the Literature

**DOI:** 10.7759/cureus.55547

**Published:** 2024-03-05

**Authors:** Nikola Stojanovic, Ugochukwu J Ebubechukwu, Michael Schaible, Asher Gorantla, Inna Bukharovich

**Affiliations:** 1 Internal Medicine, Brookdale University Hospital Medical Center, Brooklyn, USA; 2 Internal Medicine, SUNY (State University of New York) Downstate Medical Center, Brooklyn, USA; 3 Cardiology, SUNY (State University of New York) Downstate Medical Center, Brooklyn, USA; 4 Cardiology, Kings County Hospital Center, Brooklyn, USA

**Keywords:** intravenous immunoglobulins (ivig), aspirin therapy, acetylsalicylic acid, skin peeling, strawberry tongue, high fever, kawasaki disease treatment, kawasaki disease in atypical age group, incomplete kawasaki, kawasaki disease

## Abstract

This case report describes a 21-year-old female who was diagnosed with Kawasaki disease (KD), a rare condition in adults. Careful clinical assessment, including the history of a recent upper respiratory tract infection and the physical findings of fever, sinus tachycardia, strawberry tongue, and skin peeling of the hands and feet, prompted further evaluation. Laboratory findings supported an inflammatory process, and multidisciplinary consultations led to the diagnosis of KD. Prompt treatment with acetylsalicylic acid and intravenous immunoglobulin resulted in rapid improvement and prevention of the severe complications associated with untreated KD, particularly in the cardiovascular system. This case emphasizes the importance of the high risk of suspicion and the need for a comprehensive evaluation in atypical presentations of KD in adults, where early recognition and management are crucial to prevent long-term sequelae such as coronary artery aneurysms and myocardial infarction.

## Introduction

Kawasaki disease (KD) is an acute inflammatory disorder that affects small and medium-sized arteries, especially coronary arteries [1]. This condition can lead to the formation of coronary aneurysms, blockages in the arteries, or, in some cases, sudden cardiac death [2]. Dr. Tomisaku Kawasaki initially described and established the diagnostic criteria for the mucocutaneous lymph node syndrome in Japan in 1967. This was later renamed KD after the author, and published in English in 1974 [1].

KD is commonly seen as a childhood disease, as up to 85% of patients are children aged five or less, and it is the second most common cause of acquired heart disease in childhood in developed countries. But KD is not an exclusive childhood disease, and according to projections made through system dynamics modeling, it is anticipated that by the year 2030, approximately 175,000 individuals in the U.S.A., or 1 out of every 1600 adults, will be affected by KD [2].

As per one Japanese study, up to 9.1% of myocardial infarctions and sudden cardiac deaths in young adults can be attributed to a previous KD and its consequences [3]. The high risk of suspicion is required in early-onset coronary ischemia, particularly in the absence of other coronary artery disease risk factors, as many of those patients lack typical anginal pain.

## Case presentation

A 21-year-old African American female patient with an unremarkable prior medical history presented with initial symptoms of bilateral ocular redness and glossitis, accompanied by bilateral palmar and plantar burning sensations, temporomandibular discomfort and pain, impeding mouth opening, and fissured lips for a week. A review of systems revealed a recent history of high-grade fever and mild substernal pressure-like chest discomfort. However, she denied having any cough, dyspnea, abdominal pain, diarrhea, or urinary symptoms. Further inquiry revealed a self-limited upper respiratory infection approximately one month before presentation. The family history was non-contributory.

Physical examination was notable for sinus tachycardia (heart rate of 119 beats per minute), blood pressure of 113/75 mmHg, rectal temperature of 39.1 degrees Celsius, bilateral conjunctivitis, strawberry tongue, bilateral interphalangeal joint swelling without tenderness, and erythema with desquamation of the palms and soles. An electrocardiogram confirmed sinus tachycardia without other abnormalities. The laboratory results are outlined in Table 1.

**Table 1:** Initial laboratory results on admission g/dL = gram per deciliter; mm3 = per 0.001 milliliter; mg/dL = miligram per deciliter; mm/h = millimeter per hour

Laboratory Parameters	Patient’s values	Reference range (females)
WBC	16,000 / mm^3^	4,500 – 11,000 / mm^3^
RBC	4,500,000 / mm^3^	3,900,000 – 5,200,000 / mm^3^
Hemoglobin	12.2 g/dL	11.6 – 14.5 g/dL
Platelet	501,000 / mm^3^	150,000 – 450,000 / mm^3^
C-reactive protein	161 mg/dL	< 1 mg/dL
Erythrocyte sedimentation rate (ESR)	111 mm/h	0 – 20 mm/h
Serum albumin	2.8 g/dL	3.4 – 5.4 g/dL

Leucocytosis was significant for neutrophil predominance and left shift. The rest of the comprehensive metabolic panel, urinalysis, pregnancy test, and urine toxicology were unremarkable.

The patient was admitted for further evaluation and management. Serological and molecular testing for parvovirus B19 (immunoglobulin M (IgM) and DNA PCR) and beta-HCG were negative. Autoimmune screening showed a positive antinuclear antibody (ANA) titer at 1:80. Still, assays for anti-streptolysin O, perinuclear-antineutrophil cytoplasmic antibodies (P-ANCA), cytoplasmic-ANCAs (C-ANCA), Sjogren's antibodies, anti-double stranded DNA, anti-Smith antibodies, and anti-SCL-70 antibodies were unremarkable. Chest radiography did not reveal any acute pathological findings. An initial echocardiogram exhibited normal systolic and diastolic function, absence of valvular anomalies, and no findings on coronary arteries. The infectious disease consult team expressed a low concern for acute infection and prompted consultation with the inpatient rheumatology and cardiology team. The multidisciplinary team concluded that the patient's condition would be best explained by incomplete, also known as atypical, Kawasaki disease in an adult. The therapeutic approach included high-dose acetylsalicylic acid (80 milligrams per kilogram of body weight per day divided into four doses a day) for five days, a single dose of intravenous immunoglobulin G (2 grams per kilogram of body weight), besides supportive intravenous hydration (normal saline at rate 84 milliliters per hour) for two days. The patient exhibited remarkable improvement within two days of treatment initiation, facilitating her discharge on the sixth day of hospitalization.

The patient presented no additional complaints and maintained consistent follow-up visits with the cardiology and rheumatology departments over the next year. Furthermore, an annual echocardiogram was performed repeatedly and showed no significant findings.

## Discussion

KD is an immune-mediated disease of the arterial wall hypothesized to be triggered by infections like Streptococci, Staphylococci, and Propionibacterium acnes. Many viruses were linked to KD, but no definitive causation was proven. Still, the exact cause of KD is unknown [4]. The hypothesis is that a culprit leads to an uncontrolled immunologic response, causing panvasculitis with a predilection for coronary arteries, usually affecting children due to immature immune systems [5]. Inflammation of the blood vessels leads to endothelial dysfunction, causing increased vascular permeability, vasoconstriction, and susceptibility to thrombogenesis. About 25% of untreated patients with KD suffer significant damage to the tunica media of the coronary arteries and subsequent aneurysmal dilatation [4]. Still, in the era of newer management options (e.g., intravenous immunoglobulin (IVIG) and high-dose acetylsalicylic acid (ASA)), only about 5% of patients with KD develop coronary aneurysms [5,6]. Later in the course, myointimal proliferation leads to intimal thickening and vascular remodeling, causing pseudonormalization of the vascular lumen with subsequent calcifications and possible stenosis formation at the outlet of the prior aneurysm [4]. Importantly, pseudonormalization of the coronary arteries does not indicate the resolution of the disease, as these patients are still at heightened risk of complications when compared to the general population [7,8].

A typical presentation follows a brief, non-specific prodrome of respiratory or gastrointestinal symptoms [9]. In children, clinical presentation typically comprises a fever of more than five days, bilateral conjunctivitis, cervical adenopathy, polymorphous skin rash, and oral mucous lesions [10].

The clinical manifestations in adults are like those in children. However, they may vary in severity and presentation, and it is thus challenging to make a diagnosis in adults due to the rarity and variability of symptoms. Younger adults can present with early-onset cardiac ischemia in the absence of preceding angina, and thus, a high-risk index of suspicion is required [11].

Diagnosis, differential diagnosis, and workup of Kawasaki disease

KD is primarily a clinical diagnosis of exclusion based on recognizing the characteristic pattern of symptoms and signs. The American Heart Association provided guidelines for diagnosing Kawasaki disease in 2017 [12], including the criteria outlined in Table 2.

**Table 2:** The American Heart Association (AHA) diagnostic criteria for Kawasaki disease McCrindle et al. (2017) [12]

Fever lasting five or more days in the duration and presence of at least four out of five clinical features
1. Changes in extremities like reddening of palms and soles, swelling of the hands and feet, or membranous desquamation of the fingertips in the convalescent phase.
2. A non-vesicular polymorphous rash usually starts as perineal erythema and desquamation, followed by macular, morbilliform, and targetoid skin lesions of the trunk and extremities. Patients may also have redness of crust formation at the site of BCG vaccination.
3. Bilateral conjunctival injection without exudate.
4. Changes in the lips and oral cavity like red, cracked lips, strawberry tongue, and/or diffuse erythema of the oral or pharyngeal mucosa.
5. Cervical lymphadenopathy with at least one lymph node measuring 1.5 cm in size and more is the least consistent feature of KD.

Although cardiovascular issues are the most severe complications in KD, they are not included in the diagnostic criteria because most patients do not develop them. The most frequent heart-related symptoms in the initial 10 days of KD constitute tachycardia and a heart gallop [12].

Classical KD is uncommon in adults, with less than 100 reported cases worldwide [13]. A 2005 review summarized major differences in clinical manifestations between adults and children in KD [14], as outlined in Table 3.

**Table 3:** The main differences in clinical manifestations of Kawasaki disease in adults and children Sève et al. (2011) [14]

Clinical manifestations	Adults	Children
Cervical adenopathy	93%	75%
Hepatitis	65%	10%
Arthralgia	61%	38%
Meningitis	10%	34%
Thrombocytosis	56%	100%
Coronary aneurysms	5%	20%

Some children and adults do not fulfill the classic criteria but can still have KD, and that is known as “incomplete or atypical” Kawasaki disease [5]. It refers to patients with fever lasting >5 days and two or three clinical criteria without other reasonable explanations of the disease. Incomplete KD in adults is even rarer, with just a dozen reported cases so far [13].

In 2004, a diverse group of specialists developed a guideline to help healthcare providers determine when a patient with a fever lasting more than five days but presenting fewer than four clinical signs of KD should have echocardiography and receive IVIG treatment [15]. The proposed criteria for incomplete KD are outlined in Table 4.

**Table 4:** The proposed criteria for incomplete, also known as atypical Kawasaki disease Newburger et al. (2004) [15]

If three or more laboratory criteria are present, the patient should have echocardiography and be treated for Kawasaki disease
Serum albumin < 3 g/dL
Anemia
Elevation of the liver function tests
Platelet counts > 450,000/mm^3^ after 7 days of symptoms
White blood count > 15,000/mm^3^
Urinalysis showing >10 WBC/hpf

The differential diagnoses of KD include adenovirus, echovirus, measles, group A Streptococcus infection (e.g., scarlet fever and toxic shock syndrome), Rocky Mountain spotted fever, leptospirosis, drug reactions, Steven-Johnson syndrome, and serum sickness.

For all patients with suspected KD, conducting echocardiography early to establish a baseline for ongoing monitoring and assessing treatments' effectiveness is essential [12]. Understanding variations in coronary artery sizes across different genders and age groups has enhanced the role of coronary artery (CA) diameter measurements, which can be particularly useful in deciding whether patients who do not meet the classic KD diagnostic criteria should still receive IVIG treatment. Echocardiography is also crucial for detecting coronary aneurysms, ectasia in the arteries, reduced heart muscle contractility, and pericardial effusions. The 2017 American Heart Association (AHA) guideline for managing Kawasaki disease stresses the importance of regular echocardiography follow-up because of its accessibility and reliability [15]. Recent advancements in non-invasive techniques have introduced magnetic resonance coronary angiography (MRCA) and multiple detectors computed tomography (MDCT). MDCT provides enhanced image resolution, whereas MRCA offers the benefit of avoiding radiation exposure [12].

Management of Kawasaki disease

The foundational recommendations for treating both complete and incomplete Kawasaki disease are outlined in the 2017 guidelines from the American Heart Association. The treatment approach for complete and incomplete KD is the same, acknowledging that patients with incomplete KD may not meet all the criteria for a complete diagnosis but would still benefit from the standardized treatment protocols [15].

Administering intravenous immunoglobulin (IVIG) within the first 10 days of the illness can decrease the occurrence of coronary artery aneurysms by up to five times compared to children who do not receive it, making IVIG a fundamental part of acute KD treatment. The IVIG is used at a dose of 2 g/kg as a single infusion during the 10-to-12-hour infusion, and the successful clinical response is considered the disappearance of fever. It is reasonable to administer IVIG even after the 10-day window of acute illness if the patient still experiences fever and has high inflammatory markers. The erythrocyte sedimentation rate (ESR) is affected by the administration of IVIG and should not be used to assess response to IVIG [15].

ASA is used at a moderate (50 to 80 mg/kg/day divided into four doses per day) or high dose range (80 to 100 mg/kg/day divided into four doses per day) primarily due to its potent anti-inflammatory, antipyretic, and anti-platelet effects in the acute phase of KD. High-dose ASA is a reasonable option until the patient is afebrile. Combining clopidogrel with ASA has proven superior to using either medication in reducing vascular events within both the coronary and cerebral arteries in adults. Another anti-platelet agent, dipyridamole, besides ASA and clopidogrel, has been studied and used [15].

Every one in five patients treated with IVIG will either have a recurrence of fever or persistence of fever after a single infusion, and it is considered IVIG resistance [15]. In cases of IVIG resistance, the most common approach would be re-administering IVIG at the usual dose with or without corticosteroid infusion (such as prednisolone 2 mg/kg/day divided into three doses a day and followed by taper until CRP normalized). The other studied management options for treating IVIG resistance include administering infliximab (a monoclonal antibody against TNF-alpha), cyclosporine, anakinra, cyclophosphamide, or even plasma exchange as outlined by the 2017 AHA guideline [15].

For cases with giant coronary artery aneurysms (diameter equal to or larger than 8 mm) or thrombosis, anticoagulation therapy with warfarin or low molecular weight heparin is advised. Combining anticoagulants and anti-platelet agents is recommended for giant aneurysms to minimize thrombosis risk, as using anticoagulants alone has a higher chance of vessel blockage and myocardial infarction. Statins could be beneficial for some patients to improve endothelial dysfunction, but they are not considered the standard of therapy and require more research [15].

If any symptoms of myocardial ischemia or imaging studies are positive for high-risk aneurysms, the next step should be coronary angiography with possible percutaneous coronary intervention (PCI). In individuals with KD complications, the impacted coronary arteries are prone to accumulating a significant amount of thrombus [15]. This often necessitates the use of a thrombectomy or a rotational atherectomy catheter before PCI can be successfully performed [16].

After the acute phase of the illness, the patient should be regularly followed by a cardiologist and undergo repeated echocardiography to rule out cardiovascular complications. Dual antiplatelet therapy might be considered in certain situations if risks for cardiovascular complications are deemed high. There is no consensus on the right time to stop anti-platelet therapy, as normalizing aneurysms does not eliminate the risk of future heart issues [15].

The patient from the case report did not fulfill the classic KD criteria, as she presented with a high fever and only three of the four necessary criteria: conjunctivitis, mucositis, and skin changes in extremities. However, her lab results indicated incomplete KD, characterized by leukocytosis (WBC count of 16,000/mm^3^), thrombocytosis (platelet count of 501,000/mm^3^), and slightly low serum albumin levels (2.8 g/dL). These findings facilitated a timely and accurate diagnosis, leading to immediate treatment. The peeling skin on her hands and feet, coupled with the rapid improvement of symptoms following the start of IVIG and ASA therapy, suggest she may have been entering the convalescent phase of KD.

## Conclusions

In this case report, we detailed the clinical presentation of a young female who experienced a high-grade fever, flu-like symptoms, mucositis, conjunctivitis, and skin desquamation following a self-limiting upper respiratory tract infection a month earlier, accompanied by laboratory findings indicative of an inflammatory process. Further investigation for common infectious and autoimmune conditions was unrevealing. The involvement of a multidisciplinary approach and a high index of suspicion led to the correct diagnosis of atypical, also known as incomplete Kawasaki disease, a rare occurrence in adult patients. This case report underlines the significance of recognizing that patients who do not exhibit all required criteria for complete, also known as typical Kawasaki disease, might fit in the clinical spectrum of atypical Kawasaki disease and would greatly benefit from the standard treatment of Kawasaki disease with high-dose acetylsalicylic acid and IVIG as per the American Heart Association guideline, as our patient did. The importance of timely recognition and prompt treatment of KD lies in minimizing complication risks, such as severe cardiovascular events.
